# Migration background is associated with caries in Viennese school children, even if parents have received a higher education

**DOI:** 10.1186/1472-6831-14-51

**Published:** 2014-05-09

**Authors:** Barbara Cvikl, Gertraud Haubenberger-Praml, Petra Drabo, Michael Hagmann, Reinhard Gruber, Andreas Moritz, Andrea Nell

**Affiliations:** 1 Department of Conservative Dentistry & Periodontology, Medical University of Vienna, Vienna, Austria; 2 Department of Preventive, Restorative and Pediatric Dentistry, School of Dental Medicine, University of Bern, Bern, Switzerland; 3 Laboratory of Oral Cell Biology, School of Dental Medicine, University of Bern, Bern, Switzerland; 4 Section for Medical Statistics, Center for Medical Statistics, Informatics, and Intelligent Systems, Medical University of Vienna, Vienna, Austria

**Keywords:** Vienna, Caries, DMFT, Education level, Migration background, School type

## Abstract

**Background:**

A low level of education and the migration background of parents are associated with the development of caries in children. The aim of this study was to evaluate whether a higher educational level of parents can overcome risks for the development of caries in immigrants in Vienna, Austria.

**Methods:**

The educational level of the parents, the school type, and the caries status of 736 randomly selected twelve-year-old children with and without migration background was determined in this cross sectional study. In children attending school in Vienna the decayed, missing, and filled teeth (DMFT) index was determined. For statistical analysis, a mixed negative-binomial-model was used.

**Results:**

The caries status of the children with migration background was significantly worse compared to that of the native Viennese population. A significant interaction was found between migration background and the educational level of the parents (p = 0.045). No interaction was found between the school type and either the migration background (p = 0.220) or the education level of the parents (p = 0.08). In parents with a higher scholarly education level, migration background (p < 0.01) and school type (p = 0.018) showed an association with DMFT values. In parents with a low education level, however, migration background and school type had no significant association with DMFT values.

**Conclusion:**

These data indicate that children with a migration background are at higher risk to acquire caries than other Viennese children, even when the parents have received a higher education.

## Background

Dental caries is a global health problem. The prevalence varies across countries [1-4]. Public health initiatives, oral health education and prevention programs have led to a substantial decrease in the prevalence of caries [5,6]. Industrialized Western countries have reached the European Goals for Oral Health of the WHO for 2020, which are that children at the age of twelve should have no more than an average of 1.5 DMFT (decayed, missing, filled teeth) [7,8]. However, industrialized countries are multicultural migration regions, where children with a migration background usually fail to reach the European Goals for Oral Health, as, for example, in Spain [9], Italy [10,11], Germany [12], Sweden [13,14], Denmark [15] and Greece [16]. To improve caries prevention programs, it is important to understand the risk factors for children with a migration background and why they have more caries compared to native-born children.

One of the risk factor for caries development in children is the educational level of parents. Data are available for Stockholm [17], Palermo [18], Brussels [19] and nationwide in Poland [20] and in Denmark [21]. Furthermore, the educational level of parents is also a predictor of caries in areas outside of Europe, such as Hong Kong [22], Brazil [23] and Iran [24]. Thus, the educational level of the parents is associated with the oral health of their children. School type is another risk factor for the development of caries in children. For example, in Italy, caries distribution was related to the type of school attended [25]. In Istanbul, public-school students have been shown to have more caries than children in private schools [26]. Additionally, type of school has been shown to predict caries status in Brazil [27] and Jordan [28]. Overall, the educational level of parents and children’s school type can be considered risk factors for the development of caries.

Twenty-three per cent of Vienna’s population is made up of foreigners, mainly from Eastern Europe. Another major group of immigrants were born in Turkey and in other areas of Asia [29]. Dental check-ups and treatment for all Austrian citizens are covered by national health insurance [30]. Does the education level of parents with a migration background therefore have an association with the caries levels of their children? The objective of this study was to evaluate the influence of migration background on the DMFT, including the educational level of parents and their children’s school type.

## Methods

### Study population

Our cross-sectional study was conducted between 2007 and 2008 on twelve-year-old school children in Vienna. The ethics committee of the Medical University of Vienna approved the study. The ethics committee did not request an ethics commission number. The epidemiological investigation was carried out after informed consent was obtained. Schools all over Vienna were contacted, whereupon 117 agreed to participate. Cooperating schools were divided according to their location in Vienna (by district). Schools of two different types were randomly selected from each district. School type 1, is an academic-level high school mostly preparing the students for higher education at a university that offers education/level 2A according to the International Standard Classification of Education. School type 2 is an elementary-level school that offers lower secondary education/level 2C according to the International Standard Classification of Education and prepares children for direct access to the labor market [31].

The purpose of randomization in each district was to keep the socio-economic influence of the school area as small as possible. In each school, an average of 19 (18.9 +/− 4.4) children were examined, and they were matched according to their migration background. Equal numbers of children with and without migration background were not enrolled in each individual school; however, this was compensated for in another school of the same district. Immigrant status was assigned if the child or at least one parent was not born in Austria (persons with foreign background) [32]. Non-migration background was assigned if the child and both parents were born in Austria.

### Clinical examination

After obtaining informed consent from the parents, clinical examinations were performed in the schools with a dental probe and a mouth mirror under natural light, according to the recommendations of the World Health Organization (WHO) for epidemiological surveys [33]. The same investigator, who had been specially trained and calibrated, clinically examined the schoolchildren. The time spent on the calibration process (theoretical discussions, training, and calibration exercises) was two days. Furthermore, children of one school were examined under supervision before the study started. To assess the dental status and caries experience of the children, the DMFT and DMFS indices (WHO 1997 criteria) were used whereby only permanent dentition was included in the study. Decayed (D), missing (M) and filled (F) teeth (T) or tooth surfaces (S) were counted in every child. Missing teeth were counted only if they were missing as a consequence of caries. The SiC Index (Significant Caries Index), the mean DMFT of the one-third of the group with the highest DMFT scores, and the necessity of treatment due to dental decay (DT score) were subsequently calculated using those values. The indices were additionally used in this study, as the mean DMFT value does not correctly reflect the distribution, leaving high caries groups undiscovered in the population. The SiC Index, for example, focuses on thirty per cent of a certain group of people or population with the highest caries scores. The index includes the frequently skewed distribution of caries in the population and is also used for oral health goals [34]. The European Goal for Oral Health is that the SiC Index should be less than 3 among 12-year-olds by the year 2015 [35].

### Questionnaire

The migration background of children and parents, the highest parental level of education (mother or father) and former dental treatment and experiences were recorded by parents in a questionnaire. Regarding parents’ educational level, a distinction was made between low educational level (no education or compulsory schooling) and high/medium educational level (apprenticeship training, vocational school, high school or higher education).

### Statistical analysis

The data were analyzed using SPSS version 17.0 (SPSS Inc., Chicago, Ill., USA) and SAS 9.3. (SAS Institute Inc., North Carolina, USA). An ANOVA model applying Tukey’s post-hoc test was used for normally-distributed data*.* The Kruskal Wallis test was used in cases in which data were not normally distributed. The influence of migration background, type of school and parents’ educational level on DMFT was modeled using a negative binomial mixed model taking into account the highly right-skewed variables. This model includes all three variables plus all possible two way interactions. If an interaction term that includes the educational level of the parents turned out to be significant, separate models were built for this variable. To estimate final effects, insignificant interaction terms were removed from the models, which were then recalculated. Least squares estimates for the IRR (Incidence Rate Ratio) for these models are provided. P values less than 0.05 were judged to be significant.

## Results

### Study population

Among the 736 children who were examined, 50.7% had a migration background, and 49.3% were native Austrians (Table 1). Males and females constituted 44.3% and 55.7% of the sample, respectively. Of these children, 48.2% attended school type 1 and 51.7% attended school type 2. Of the parents 83.4% had a medium/high educational level, as stated in the questionnaire and 16.6% had a low educational level, respectively. The participation rate of children who had agreed in advance to participate in the study and were selected was 100%. However, it is unknown how many children or parents refused to participate in the study in advance, as that decision was made in the schools.

**Table 1:** Study population

	**Education level of the parents**			
	**Low**		**High/medium**	
	**Migration background**		**Migration background**	
	**Yes**	**No**	**Yes**	**No**
**School type**				
I (Level 2A)	**16**	**6**	**117**	**216**
II (Level 2C)	**83**	**17**	**157**	**124**

### Basic caries status of the children

The results of DMFT, DMFS, SiC index, DT and MT are presented in Table 2 in means (plus SD) and level of significance, based on a univariable analysis. Overall, caries status was worse in children with a migration background compared to children without migration background. The mean DMFT index was 2.33 and 1.50 (p < 0.001), the mean DMFS index was 3.51 and 2.15 (p < 0.001), the mean SiC index was 4.93 and 3.54 (p < 0.001), respectively among children with a migration background and children without migration background. The need for treatment was 2.4 times higher in children with a migration background compared to children without migration background, demonstrated by the mean DT of 0.83 and 0.34, respectively (p < 0.001). Tooth loss as a result of caries was observed 3.5 times more frequently in children with a migration background compared to children without migration background as shown by the reflecting MT values of 0.07 and 0.02, respectively (p = 0.010).

**Table 2:** Oral health status

**Variables**	**Children with migration background (n = 373)**	**Children without migration background (n = 363)**	**p-value**
**DMFT**	2.33 (2.28)	1.50 (1.77)	< 0.001
**DMFS**	3.51 (4.10)	2.15 (2.80)	< 0.001
**SiC**	4.93 (1.70)	3.54 (1.50)	< 0.001
**DT**	0.83 (0.14)	0.34 (0.80)	< 0.001
**MT**	0.07 (0.30)	0.02 (0.10)	0.010

Comparison of the DMFT (decayed, missed and filled teeth), DMFS (decayed, missed and filled surface), SiC (significant caries) index and between DT (decayed teeth) and MT (missed teeth) among children with migration background and children without migration background.

Data are expressed in mean (plus SD) and level of significance.

### Association with education level of parents and caries status of the children

When considering DMFT as the main target variable, a significant interaction was found between the migration background and the educational level of parents (p = 0.045). The mean DMFT index was 2.33 and 1.50, respectively among children with and without migration background. Regarding the educational level, the mean DMFT index was 2.18 and 1.87, respectively, among low and high/medium educational level. These data suggest that migration background as well as the educational level of parents affects the caries status of their children. No interaction was found between school types and migration background (p = 0.219) or the educational level of parents (p = 0.079). For further analysis it was distinguished between children of the group “parents with high/medium educational level” and children of the group “parents with a low educational level”.

In parents with high/medium educational level no interaction was found between migration background and school types (p = 0.227). The data suggest that the two variables are independent, and interaction was removed from the model. In a new analysis, migration background (p < 0.001) and school type (p = 0.018) had a significant association with DMFT values. A significant IRR of 0.787 comparing children attending school type 1 (level 2A) versus those attending school type 2 (level 2C) was observed. Therefore a 22% lower DMFT count for children attending school type 1 compared to those attending school type 2 was expected, when all other variables are kept constant. Furthermore, a significant IRR of 1.52 comparing children with no migration background with those with migration background was observed. Thus, a 52% higher DMFT count for children with migration background was expected, given that all other variables are kept constant (Table 3). These data suggest that children with a migration background are at higher risk of acquiring caries than the native born population even if their parents have a higher educational level. The children’s school type is a risk factor for caries, however, but less than “migration”.

**Table 3:** Least squares (parents with a high/medium education level)

**Least squares**				
	**IRR**	**low95**	**up95**	**p**
School type (2A vs. 2C)	0.787	0.6459	0.9589	0.0178
migration background (yes vs.no)	1.5249	1.2765	1.8216	<.0001

Migration background and school type had a significant association with DMFT values in children of parents with a high/medium education level.

In parents with a low educational level, no interaction was found between migration background and school type (p = 0.875) In this sub-population, migration background (p = 0.863) and school type (p = 0.491) have no significant association with DMFT values (Table 4). The mean DMFT index was 2.16 and 2.26, respectively among children of low educated parents with and without migration background. Regarding the school type, the mean DMFT index was 2.50 and 2.26, respectively among school type 1 and 2.

**Table 4:** Least squares (parents with a low education level)

**Least squares**				
	**IRR**	**low95**	**up95**	**p**
School type (2A vs. 2C)	1.2066	0.7086	2.0547	0.4909
migration background (yes vs.no)	0.954	0.5602	1.6246	0.8626

Migration background and school type had no significant association with DMFT values in children of parents with a low education level.

## Discussion

One main finding of this study was that in parents with a higher educational level, migration background and school type have an association with DMFT values. Hitherto unnoticed, in parents with a low educational level, however, migration background and school type have no significant association with DMFT values. These results suggest that children of parents with a migration background are at higher risk of acquiring caries than other children in Vienna, even if parents of the former have a “higher educational level” of apprenticeship training or vocational school education.

The results indicate that children with migration background, attending school in Vienna, would need more support for achieving efficient oral health, even if their parents have received a medium or high educational level. Possible explanations might be the socio-economic status, language barriers, social standing, dietary habits and other factors which were not addressed by this study. The oral health knowledge children receive at school can at least partially compensate for the impact of family oral hygiene. Consequently, in particular children with a migration background, attending school type 2 (level 2C), would benefit from specifically designed oral health care programs.

Our findings are in agreement with epidemiologic studies reporting on the prevalence of caries in school children with migration background in Spain [9], Italy [11], Germany [12], Sweden [13] and Denmark [15]. Furthermore, our findings support reports that the educational level of parents is associated with the prevalence of caries in children, although children in the other studies were younger. Most studies only took the educational level of mothers into account [18,20,23]. Interestingly, an increased risk of caries was associated not only with low educational levels of mothers but also even with their high acquisition of university degrees [36]. We found that even when parents have a higher scholarly education level, migration background has an association with DMFT values. When parents have low educational levels, migration background and school types are not associated with caries. The results can neither be confirmed nor disproved by other studies since comparable studies combining the variables migration background, high and low educational levels of parents and school types of children are not available to our knowledge. However, the results of our study are in line with studies combining migration background and educational levels [21].

Interestingly, children with migration background in families with highly-educated parents have poorer dental health status than others. Culturally-influenced dietary habits may explain these findings. Living conditions in the country of origin often affect behavior in the country of migration [37,38]. This hypothesis is supported by others [39]. However, those studies also considered the financial background of immigrant families and their length of stay in their new countries One major conclusion of the present study is that even highly-educated immigrant parents need to be informed about the risk of caries development in, and prophylaxis for, their school-aged children.

Together, our findings highlight the need for healthcare programs even for more highly-educated immigrants. Particular interest should also be drawn to special caries prevention programs and dental health education programs. Teeth-brushing programs, educational work, distribution of flyers and lectures on dental hygiene should not be restricted to schools and kindergartens; rather, they should also be provided by religious centers and cultural establishments where parents and children can participate together, in programs [40]. In addition, regularly-performed dental checkups of all Austrian citizens might help sensitize immigrants and parents with low educational levels to the oral health of their children. Dental checkups, for example, could be provided by the mother-child-pass examination, an Austrian health program. Especially early oral healthcare promotion might improve children’s oral health regardless of their origin [41]. Parents of native Austrian children with low educational levels might also benefit from such healthcare strategies.

One limiting factor of this study may be that the oral health care of participating children may be better than that of non-participating children. In this study, children and their parents were asked by schools to participate and were nominated for the study when they agreed. Although the participation rate of nominated children was 100%, it is unknown how many children did not agree to participate in the study in advance. Fear of dental examinations or embarrassment over poor teeth may have discouraged participation in the study. Furthermore, owing to one of the main focuses to distinguish between children of parents who have completed compulsory education only, and of parents with a vocational training or higher education, it was not possible to gain the same numbers of participants in all groups. Another bias might be the correlation between school types attended by children and the socio-economic status of their families. We tried to minimize that bias by selecting schools from all of Vienna districts, because life-style is usually a good indication of socio-economic status.

In future studies, the birth countries of immigrant children should be taken into account by comparison the DMFT scores of children still living in the countries and children that were born in the countries, now living in Vienna. Furthermore the knowledge about the children’s original countries could provide more sensitive data, since the prevalence for caries might be different in the countries of origin. In this way one could figure out the group of children with the highest risk and support them and their parents accordingly. It would also be interesting to find out if there is a difference in oral health between children with migration backgrounds born in Austria and those born abroad. Another point of consideration is determination of how long immigrant families have been living in Austria. This would be of particular interest, since in the present study no information is given about current and previous risk of the children, because it was not able to investigate whether the children with migration background, not born in Austria, got caries in Austria or in their home country.

The education of parents, used to measure socio-economic status, reflects the material, intellectual and other resources of their families of origin. Moreover, higher educational levels may make people more receptive to health-education programs [42]. This is partially in contrast to the findings of this study that children of parents with a migration background are at higher risk of acquiring caries, even if parents have a “higher educational level”. Consequently, in this study, the higher education of the parents do not compensate for the increased caries susceptibility of their children. Therefore, several factors should also be considered to have an influence on the development of caries in children like e.g. genetic and biological factors, social environment, physical environment, health behavior, dental and medical care and time, as described in a conceptual model by Fisher-Owens et al. [40]. Whether these factors have different influence in children regarding their country of origin cannot be answered with the results of this study, but should be considered in the planning of future studies.

## Conclusion

The most relevant finding of this investigation is that migration background is a main risk factor for the occurrence of caries in Viennese school children, even if their parents have received at least a basic scholarly education. Caries-prevention programs, oral-health education and motivation would be helpful, especially for children and parents with a migration background, regardless of the latter’s educational level.

## Competing interest

The authors declare no potential conflicts of interest with respect to the authorship and/or publication of this article.

## Authors’ contributions

BC: Analyzed the data, wrote the paper. GHP and PD: Performed the clinical examination. MH: Performed statistical analyses, contributed to review process. RG: Wrote the paper, contributed to discussion. AM and AN: Contributed to discussion. All authors read and approved the final manuscript.

## Pre-publication history

The pre-publication history for this paper can be accessed here:

http://www.biomedcentral.com/1472-6831/14/51/prepub
